# Scheme of Study for the College of Dental Surgery, University of Michigan

**Published:** 1891-03

**Authors:** 


### Scheme of Study for the College of Dental Surgery University of Michigan.

FIRST YEAR.

| First Semester: | Hours each week. |
|---|---|
| Osteology | 3 |
| Materia Medica | 3 |
| Analytical Chemistry and Lectures | 18 |
| Dental Laboratory Practice | 10 |
| Lectures on Prosthetic Dentistry | 1 |
| | 35 |

| Second Semester: | Hours each week. |
| --- | --- |
| Descriptive Anatomy | 3 |
| Specian Dental and Practical Anatomy | 18 |
| Materia Medica | 3 |
| Dental Laboratory Practice | 10 |
| Lectures on Prosthetic Dentistry | 1 |
| | 35 |

SECOND YEAR.

| First Semester: | |
| --- | --- |
| Descriptive Anatomy | 2 |
| Comparative Dental Anatomy | 2 |
| Histology, Lectures and Laboratory work | 9 |
| Bacteriology | 3 |
| Prosthetic Dentistry | 16 |
| Operative Dentistry | 3 |
| | 35 |

| Second Semester: | |
| --- | --- |
| Physiology | 3 |
| Physiological Chemistry | 3 |
| Organic Chemistry | 3 |
| Prosthetic Dentistry | 16 |
| Operative Dentistry | 3 |
| | 28 |

ENTIRE THIRD YEAR.

| | |
| --- | --- |
| Operative Dentistry | 3 |
| Clinical Operative Dentistry | 15 |
| Oral Pathology and Surgery | 2 |
| Dental Materia Medica and Therapeutics | 2 |
| Laboratory practice in crown and bridge work, construction of regulating devices, instruments and appliances for Clinical Dentistry | 10 |
| | 32 |

